# Crime and victimisation in people with intellectual disability: a case linkage study

**DOI:** 10.1186/s12888-016-0869-7

**Published:** 2016-05-28

**Authors:** Billy C. Fogden, Stuart D. M. Thomas, Michael Daffern, James R. P. Ogloff

**Affiliations:** Faculty of Social Sciences, University of Wollongong, New South Wales, 2522 Australia; School of Global, Urban and Social Studies, RMIT University, Building 37, Level 4, Swanston Street, Melbourne, Victoria 3001 Australia; Centre for Forensic Behavioural Science, Swinburne University of Technology, Melbourne, Australia; Southern Clinical School, Monash University, Melbourne, Australia

**Keywords:** Intellectual disability, Victimisation, Offending, Mental disorder

## Abstract

**Background:**

Studies have suggested that people with intellectual disability are disproportionately involved in crime both as perpetrators and victims.

**Method:**

A case linkage design used three Australian contact-level databases, from disability services, public mental health services and police records. Rates of contact, and official records of victimisation and criminal charges were compared to those in a community sample without intellectual disability.

**Results:**

Although people with intellectual disability were significantly less likely to have an official record of victimisation and offending overall, their rates of violent and sexual victimisation and offending were significantly higher. The presence of comorbid mental illness considerably increased the likelihood of victimisation and offending; several sex differences were also noted.

**Conclusions:**

People with intellectual disability are at increased risk for both violent and sexual victimisation and offending. The presence of comorbid mental illness aggravates the risk of offending and victimisation. Future research should focus on a more nuanced exploration of the risks associated with intellectual disability and specific mental disorders and related indices of complexity.

## Background

People with an intellectual disability (ID) are a marginalised and vulnerable group. The available research suggests an association between ID and criminal offending [1–3]; this has served to propel public fear and reinforce perceptions of the need for social distance. However, the evidence from which these conclusions have been drawn remains far from definitive, with significant methodological limitations marring what are arguably tentative conclusions [1, 2, 4]. A related area that has received much less scientific attention is criminal victimisation, despite a compelling argument that specific deficits in interpersonal functioning and cognitive capability potentially increase exposure to dangerous situations, therefore contributing to the likelihood of criminal victimisation [5–14].

### Intellectual disability and criminal victimisation

Intellectual Disability is characterised by significant impairments in intellectual functioning alongside difficulties in daily tasks, personal responsibility and communication [15, 16]. From a theoretical standpoint, Routine Activities Theory [17] conceptualises victimisation in relation to an interaction between an available victim, the absence of a capable guardian, and a motivated offender. It reasons that people with similar lifestyles or routine activities face similar victimisation risks as they are exposed to risky places and potential offenders [5]. Some research has suggested that people with ID are most commonly victimised by their carers [5]. Routine Activities Theory would argue that victims are easily accessible in their home / living environment (availability) and there is less protection of the victim if the perpetrator *is* the guardian (absence of a capable guardian); the carer offender may be motivated to offend due to carer stress, a provocative or frustrating incident, in this context offending may be facilitated by increased potential to evade prosecution (motivated offender). A study by Sobsey [18] supports this proposition and demonstrates the substantial vulnerability of victims with ID, noting that 44 % of perpetrators had contact with a victim through disability services, in which the victim was in close proximity with the perpetrator and was less likely to recognise or report a crime due to the apparent legitimacy of the disability service. As such, victimisation can be seen as a product of complex interactions between the environment, the victim(s) and the perpetrator(s).

The available research is consistent with this theoretical stance, noting increased rates of victimisation among people with ID compared to the general population. Wilson and Brewer [6] estimated that a diagnosis of ID doubled the risk for victimisation and vastly increased the likelihood of sexual assault and being the victim of robbery compared to the general community. Other studies report similar findings, estimating that the risk of victimisation is between three and seven times higher in people with ID compared to the general community [11, 13, 19]. Of particular concern are the high rates of sexual victimisation [19–22] and the finding that the majority of crimes were committed multiple times [23], across a range of ages and environments [13, 24]. Of note, these victimisation experiences are rarely reported to authorities [6, 7, 19, 23].

Surprisingly, there is little knowledge on the relationship between gender and victimisation among people with ID. While there is some evidence from general population estimates implicating males as being more vulnerable to victimisation [25], it is not known whether this trend exists in the ID population. That being said, tentative conclusions can be drawn from general disability research, which suggests that females are more susceptible to violent and sexual victimisation compared to males [20, 26]. In sum, the available literature suggests that people with ID are at a greater risk of victimisation compared to non-disabled members of the general public, however it is far from a robust conclusion. This is for two reasons; first, of the few studies conducted, too many focus on outdated retrospective self-report data, which rely on the individual recalling events and judging whether a crime was committed. This presents a difficulty with people who have an ID as there are often disruptions in their memory functioning and judgements about their own, and the activities of others, when recalling crimes [7], consequently past research may either under report or over report actual rates of victimisation. Second, the operational definition of ID varies between studies and importantly, samples tend to be small and lack community comparison groups; both of which would arguably help contextualise the nature, direction and extent of victimisation [10].

### Intellectual disability and offending

Like victimisation, the functional deficits evident in ID suggest that people with ID may also be likely to offend. This sentiment has a long tradition [27], attracting consistent research attention over the years; with studies claiming that people with ID are overrepresented among individuals processed by the criminal justice system [28–30]. The estimated prevalence of offending in people with intellectual disability ranges from two to ten per cent and varies depending on the population and methods utilised [27, 31]. There is much variation within prison populations, with estimates ranging from less than 2 % to as high as 30 % [29, 30], yet there is little agreement on a standardised conceptual definition of what criminal offending is across these studies. A recent systematic review, pooling results from ten studies and including a total of 11,969 prisoners concluded that typically 0.5 % to 1.5 % of prisoners are diagnosed with intellectual disabilities [32]. Estimating offending prevalence with prison populations is problematic as many individuals with ID have been diverted into the community or forensic services rather than prison, so there may be an under-estimation of the true prevalence using this method.

Court appearances and police contacts provide an alternative means of establishing prevalence and are more sensitive, as these records more adequately capture the extent of contact people have with the criminal justice system. The available literature at this interface estimates that around 1 in 10 people with ID will come into contact with the police or courts as a perpetrator of crime [14, 33]. These rates are substantially different to those in the general population, with males with ID being three times more likely than males in the general public to have a prior conviction, while females have been found to be four times more likely to have a prior conviction. Interestingly, this figure was more pronounced for violent offences, with males four times higher and females 25 times higher, therefore potentially suggesting a significant vulnerability to violent offending among people with ID [34].

Some evidence suggests that people with ID are susceptible to the perpetration of specific crimes, such as sexual offences [2]. Further, there are additional factors that potentially complicate the hypothesised link between ID and offending, with findings revealing that complexities such as childhood neglect, physical health problems, mental health problems and perinatal adversity are particularly common among offenders with ID. There is also some suggestion that offenders with ID may be less effective at evading police and more visible as perpetrators [5] and this is the reason for increased prevalence rates.

The relationship between crime perpetration and ID and mental illness, which is highly comorbid with ID [33], has received empirical scrutiny. Hodgins and others [34] estimated that the presence of mental illness increased offending by five times in psychiatric inpatients compared to those with ID who had not been admitted for mental health treatment. Additionally, Vanny [33] found that nearly half of those people with ID who were referred to court had a mental illness, thereby suggesting a more complex group who may be at increased risk of criminal offending.

### Aims and hypotheses

Against this background, this study sought to determine the prevalence of criminal victimisation and offending in an ID population and to compare this to a sample of people drawn from the general population. Based on the findings of Wilson and Brewer [6] it was hypothesised that people with ID would have higher rates of victimisation and offending relative to the community sample. Secondly, based on the findings by Sobsey [19], sexual crimes were expected to be increasingly more common in the ID group. Thirdly, the added complexity brought about by having comorbid mental illness [34] was hypothesised to increase the risks of victimisation and offending above that observed in people with ID only. Finally, males with ID were hypothesized to be at greater risk of both victimisation and offending than females with ID.

## Method

### Design

The study employed a case linkage design to compare rates of criminal victimisation and offending (operationalized as criminal charges) between those with a diagnosis of intellectual disability and a community comparison sample. The research complied with contemporary Australian National Health and Medical Research Committee (NHMRC) guidelines for conducting epidemiological research; administrative permission was granted for access to data stored on contact-based databases (see below).

### Databases

Participant-level data were gathered through three archived databases; the Restrictive Intervention Data System (RIDS), the Victorian Psychiatric Case Register (VPCR) and the Victoria Police Law Enforcement Assistance Program (LEAP). The RIDS database is a state wide reporting system for individuals with a disability who have received a routine, pro re nata, or emergency restrictive intervention as defined in the *Disability Act 2006 (Vic)*. Section 3 of the *Act* defines ID as the concurrent existence of a significant sub-average general intellectual functioning, and significant deficits in adaptive behaviour, both which become manifest before the age of 18 years. Under the *Act*, a restrictive intervention can include chemical restraint, mechanical restraint or seclusion; these are mandatorily reported to Disability Services and recorded on the RIDS database and can only be used when they represent the least restrictive option. Episodes are updated monthly; individuals may only have a record of a single restrictive intervention or may be subject to repeated incidents of restrictive interventions over time. Each individual on the RIDS system has a unique client identifier. The RIDS database contains data drawn from over 150 government disability institutions across Victoria, Australia. All individuals included on the database from 1 July 2007 up till the end of 2012 were eligible for inclusion.

The VPCR (established 1961) is the state wide public mental health database in Victoria, Australia. It is a contacts-based database and documents when and why an individual comes into contact with public mental health services as well as a variety of other data including diagnostic categories, the number of contacts with services and dates and periods of admission and discharge. The VPCR records mental health diagnoses according to the International Classification for Mental Disorders version 9 and 10 (ICD 9, 10), based on thorough clinician assessments. The database does not capture contacts with primary care providers or private services so may underreport some high prevalence disorders such as anxiety, depression and substance use disorders.

The Law Enforcement Assistance Program (LEAP) database is a state wide reporting system maintained by Victoria Police that details whenever an individual has come into contact with police as a suspect, offender, victim, witness or person in need of assistance. It has been in its current format since October 1993. Incidents of offending and victimisation were extracted in raw form and, consistent with the extant literature, categorised as either: (1) violent, (2) sexual, or (3) non-violent non-sexual offences. *Violent offences* included common assault, murder, aggravated robbery, as well as any form of contact sexual offence; *sexual offences* involved indecent assault, rape and incest; and *non-sexual non-violent* offences included theft, property damage, substance misuse, threats, arson and non-contact stalking. The same three categories were used to classify both victimisation and offending histories. For the offending variables, the level of a criminal charge was selected. This point in criminal proceedings was selected in line with other recent research; a charge being indicative that there was a good deal of confidence that the alleged offence should be dealt with in a criminal court. Henceforth, for simplicity, the term offending is used to enable comparisons to be made.

### Data linkage procedure

Participant information from all databases were compiled into a single file by matching participant-level details across each database using a master list (containing full name, date of birth and gender along with a unique study ID number). Deterministic and probabilistic matching algorithms were used to maximise potential matches between databases; where individual matches were found, all relevant contact-level data were extracted. Rates of contacts and counts of criminal charges and victimisation episodes were compared to those in a random community sample from a related study of 5000 males and females drawn from the Australian Electoral Role whose case ascertainment for mental health and police contact histories had been determined using an identical methodology and the same suite of databases [35]. Due to the nature of the data available for the community sample, the offending history variable used ‘conviction’ to classify an offence history outcome for the community sample.

### Approach to analysis

Both individuals with ID and community comparison participants were compared to determine the prevalence rates of offending and victimisation in each group. The ID sample was split into two subgroups, differentiating: (1) those who had a primary diagnosis of ID and a secondary diagnosis of any mental illness (Comorbid ID group) from (2) those solely with an ID (ID only group). Prior to analysis the data were checked for missing values and a randomly selected 10 % of the ID cases (*n* = 260) were recoded and crosschecked to check for spurious data entry errors.

Variables of interest were cross-tabulated and compared using Chi Squared tests of Association, substituting Fishers Exact test statistic where cell numbers in contingency tables fell below *n* = 5. Odds ratios and relative risk statistics were then calculated, along with 95 % confidence intervals applying Miettinen’s test-based approach [36]. All associations were considered statistically significant at an alpha level of .05. Effect sizes were calculated using Cohen’s *d*, with traditional cut-offs used to determine small, medium and large effects [37]. Data were also stratified according to gender, to ascertain whether the risks for particular offence or victimisation experiences differed between males and females.

## Results

### General characteristics

The full sample comprised 2600 participants (*Males =* 1684, 64.7 %, *Females =*916, 34.2 %). The community comparison group included 4830 individuals (*M* = 2392, 49.5 %, *F* = 2438, 50.5 %). The community group were significantly older than the ID group (ID = 35.71 (16.57), community = 39.12 (12.55), *t* =9.95 (7428), *p* < .0001) with males significantly younger in the ID group (*M* = 34.12 (16.39), *F =* 38.66 (16.50), *t =* 7.13 (2911), *p* < .0001). There was no difference in age between males and females in the community group (*t =* 0.826 (4477), *p* = 0.409). Of those diagnosed with an ID, over a quarter (*N* = 709, 27.2 %) also met criteria for a comorbid mental illness and formed the Comorbid subgroup while the remaining ID sample (*N =* 1891, 72.7 %) represented the ID only subgroup.

### Victimisation

The community group were significantly more likely to have an official history of victimisation compared to the full ID sample, with the risk of victimisation being two times higher. However, at the specific crime level the rate of victimisation increased significantly for the ID sample with the rate of violent victimisation two times higher, while sexual victimisation was nearly six times higher compared to the community; effect sizes were moderate to large (Table 1). Those with ID and a comorbid mental illness had the highest rates of victimisation with a three-fold increase for violent victimisation and a ten-fold increase for sexual victimisation compared to the community. In contrast, the ID only group had higher rates of victimisation relative to the community sample, however they were victimised at a rate less than the comorbid group. Across all victimisation episodes, the comorbid ID group had approximately double the risk of being victimised compared to the ID only group (Table 2).

**Table 1:** Comparison of criminal victimisation between the total ID sample and the community sample

Victimisation Type	Total ID (*N =* 2600)	Community (*N =* 4830)	OR (95 % CI)	RR (95 % CI)	*p*	d
Any History	457 (17.5 %)	1864 (38.5 %)	0.34 (0.30–0.38)	0.46 (0.42–0.50)	<.0001	−0.59** (−0.66–-0.53)
Violent	378 (14.5 %)	314 (6.5 %)	2.44 (2.09–2.87)	2.24 (1.94–2.58)	<.0001	0.49* (0.37–0.52)
Sexual	157 (6.0 %)	49 (1.0 %)	6.27 (4.53–8.67)	5.95 (4.33–8.17)	<.0001	0.98*** (0.81–1.16)
Non Violent Non Sexual	133 (5.1 %)	1757 (36.3 %)	0.09 (0.08–0.11)	0.14 (0.12–0.17)	<.0001	- 1.33*** (−1.39–-1.22)

*Effect Sizes according to Cohen* [37] *small **medium ***large

**Table 2:** Criminal victimisation comparisons between the ID only, comorbid and community samples

Victimisation Type		OR of victimisation compared to ID only sample	OR of victimisation compared to community sample	RR compared to ID only sample	RR compared to community sample
Comorbid *N = 709*	Any History	2.15 (1.74–2.66)	0.57 (0.47–0.68)	1.84 (1.57–2.18)	0.68 (0.60–0.78)
	Violent	2.18 (1.74–2.73)	4.12 (3.34–5.09)	1.92 (1.59–2.31)	3.43 (2.88–4.08)
	Sexual	2.76 (1.99–3.82)	11.89 (8.23–17.17)	2.57 (1.90–3.47)	10.71 (7.55–15.18)
	NVNS	2.22 (1.56–3.17)	0.16 (0.12–0.21)	2.13 (1.52–2.96)	0.23 (0.18–0.29)
ID only *N = 1891*	Any History		0.27 (0.23–0.31)		0.37 (0.33–0.42)
	Violent		1.89 (1.58–2.27)		1.79 (1.52–2.11)
	Sexual		4.31 (3.00–6.18)		4.17 (2.93–5.93)
	NVNS		0.07 (0.06–0.09)		0.11 (0.09–0.13)

### Gender differences for victimisation

There were no significant differences in overall victimisation rates between males and females in the total ID group (χ^2^ = 1.67, *p* = .1962). In the community sample there were similar proportions of males and females, however the overall risk for being victimised was 1.38 times higher for males than for females (RR = 1.38, 95 % CI = 1.29–1.49).

There were substantial differences between the ID sample and the community group; the risk of violent and sexual victimisation being three and five times (respectively) higher for females with ID compared to females in the community (RR = 3.07, 95 % CI = 2.44–3.86; RR = 5.05, 95 % CI = 3.45–7.39). Males with ID were violently victimised at a rate nearly two times that of males in the community (RR = 1.76, 95 % CI = 1.47–2.10) and sexually victimised at a rate in excess of 11 times higher (RR = 11.79, 95 % CI = 6.14–22.65). Females experienced violent victimisation at a significantly higher rate compared to males in the ID sample (RR = 3.45, 95 % CI = 2.68–4.45). Sex differences according to other victimisation types were not significantly different.

### Criminal offending

Less than 10 % of the entire ID sample had a record of criminal charges according to the police LEAP database. There were no significant differences in the rates of offending between the ID sample overall (224/2600, 8.6 %) and the community sample (429/4830, 8.9 %). However, the ID sample violently offended at a rate three times higher than the community and sexually offended at a rate nearly eight times higher. By contrast, the rate of non-sexual non-violent offending was lower than that found in the community sample (Table 3).

**Table 3:** Criminal offending between the Total ID group with the community group

Offending Type	Total ID (*N =* 2600)	Community (*N =* 4830)	OR (95 % CI)	RR (95 % CI)	*p*	d
Any History	224 (8.6 %)	429 (8.9 %)	0.96 (0.82–1.14)	0.97 (0.83–1.13)	>0.05	−0.02 (−0.11–0.07)
Violent	192 (7.4 %)	119 (2.4 %)	3.16 (2.50–3.99)	3.00 (2.40–3.75)	<.0001	0.63 (0.51–0.76)
Sexual	76 (3.0 %)	18 (0.3 %)	8.05 (4.80–13.49)	7.87 (4.71–13.13)	<.0001	1.15 (0.86–1.43)
Non Violent Non Sexual	141 (5.4 %)	356 (7.4 %)	0.72 (0.59–0.88)	0.74 (0.61–0.89)	<.0001	−0.18 (−0.29–0.07)

*Effect Sizes according to Cohen* [37] *small **medium ***large

The *ID only* group violently offended at a rate 1.6 times greater, and sexually offended at a rate 3.6 times that of the community sample. The most pronounced differences were again between the comorbid sample and the community sample with the comorbid sample offending at a rate 6.5 times greater than the community for violent crimes, with this rate increasing to 18.9 times higher for sexual offences. The increased rates of offending in the comorbid group were similar even when compared to the ID only group, with a four-fold increase in violent offending and a five-fold increase in sexual offending (Table 4).

**Table 4:** Criminal offending comparisons

Offending Type		OR compared to ID only sample	OR compared to community sample	RR compared to ID only sample	RR compared to community sample
Comorbid *N = 709*	Any History	4.57 (3.44–6.06)***	2.37 (1.91–2.93)**	3.90 (3.03–5.02)	2.11 (1.77–2.52)
	Violent	4.56 (3.37–6.18)***	7.66 (5.85–10.04)***	3.93 (3.02–5.25)	6.58 (5.16–8.40)
	Sexual	5.44 (3.36–8.81)***	20.28 (11.76–34.98)***	5.13 (3.22–8.17)	18.92 (11.11–32.24)
	NVNS	6.00 (4.18–8.61)***	1.92 (1.51–2.45)**	5.33 (3.80–7.49)	1.80 (1.45–2.23)
ID only *N = 1891*	Any history		0.52 (0.41–0.65)**		0.54 (0.43–0.68)
	Violent		1.68 (1.26–2.25)*		1.65 (1.25–2.19)
	Sexual		3.73 (2.04–6.81)**		3.69 (2.03–6.71)
	NVNS		0.32 (0.24–0.44)***		0.34 (0.25–0.46)

*Effect Sizes according to Cohen* [37] *small **medium ***large

### Gender differences for offending

Among the Total ID sample, males were more likely to have a record of criminal offences compared to females (χ^2^ = 17.53, *p* < .0001), with this result also reflected in the community sample with offence convictions (χ^2^ = 196.43, *p* < .0001). Both males and females in the total ID group had significantly higher rates of criminality compared to males and females in the community group. The most pronounced difference was for females, with females with ID violently offending at a rate 11 times higher than females in the community (RR = 11.64, 95 % CI = 5.42–25.01). Males violently offended at a rate double that of males in the community (RR = 2.01, 95 % CI = 1.59–2.54) with the rate of sexual offending being nearly six times higher (RR = 5.84, 95 % CI = 3.50–9.74). Comparison of confidence intervals showed that the relative risk between the total ID group and the community was significantly higher for females than for males.

## Discussion

### Victimisation

This study investigated victimisation and offending histories in a sample of people with intellectual disability and a community comparison sample using a case linkage design. The results indicated that, overall, people with intellectual disability were less likely to have an official history of victimisation and were no more or less likely to have a history of criminal offending than people without intellectual disability. Of note, however, the ID group were significantly more vulnerable to violent and sexual victimisation *and* offending compared to the community.

These findings suggest that members of the general community are more likely to have a police record as a victim of crime overall, with the vast majority of these crimes being non-violent and non-sexual in nature. This finding is consistent with some prior research [2, 38], but contrary to previous theory and other research, which has suggested pronounced vulnerabilities for people with ID across *all* crime types [5, 6, 10, 18]. Two explanations may account for this finding. First, individuals in the ID sample may have had less exposure to certain types of victimisation experiences due to the nature of their community and/or residential living circumstances. Alternatively, non-violent non sexual crimes may be under-reported by people with ID, who may not be aware of appropriate avenues for reporting, may be unable to recognise more ambiguous non-violent non-sexual crimes, may not be progressed to police services by carers/residential staff, or that they may fear reporting a person who they depend on [5].

Baladerian and others [23] noted that less than half of violent and sexual crimes against people with ID were reported to police, and of those reported, over half said nothing happened and less than 1 in 10 perpetrators were subsequently arrested. Participants in that study cited a lack of confidence in the criminal justice system, fear of retribution and poor knowledge of reporting avenues as key barriers to reporting. The potential for underreporting should be considered. Practically, efforts to support vulnerable populations report crime should be considered.

Despite the community having a higher rate of victimisation for crime overall, the current findings demonstrate that serious offences such as violent and sexual crimes are statistically more prevalent among people with ID, which supports the study hypotheses and previous research findings [11, 13, 19–22]. Roughly 1 in 6 people in the ID sample had reported violent victimisation to police, which was twice the rate of the community; furthermore 6 % had reported sexual victimisation, which was nearly six times higher than the community rate. These figures are concerning and provide robust epidemiological insight into the extent of an under researched problem and supports the conclusions of previous smaller scale studies which noted the heightened risk for violent and sexual crimes specifically [6, 13, 19].

Of particular interest to the current study was the influence of gender on victimisation. Findings indicate that males in the community group were more likely to have records of being victims than females, consistent with prior research [25, 39–41]. Interestingly, this pattern of victimisation was not evident in the ID sample where there was no difference among victimisation types for gender, except for violent victimisation, which was significantly higher for females compared to males. This result is substantiated by the large difference in violent and sexual victimisation between the ID group and the community, for females more so than males, suggesting that females with ID are an especially vulnerable subgroup. What makes them specifically vulnerable to violent crime is unclear in the literature with only one report highlighting a possible gender difference [27]. The findings from the present study, coupled with the continued lack of consensus in the literature, should act as a catalyst to focus more on elucidating potential differences, and if so why these exist, as at present females with ID are particularly vulnerable to serious victimisation.

### Offending

The rate of criminal offending, in this sample operationalized as criminal charges being laid, was entirely consistent with previous studies [14, 33]. In this study, the overall rate of offending did not differ between the intellectual disabled and the community groups, with less than 10 % of both samples having an official record of offending. However, like victimisation, violent and sexual offending were statistically more common for people with ID, with offending six and a half times higher for violent crime and nearly 19 times higher for sexual crimes. This result can be interpreted using Routine Activities Theory [17] which postulates that a greater exposure to crime-inducing situations and personal reactions from the person with ID can make them more vulnerable to victimisation. This can also be applied to offending situations where an individual is similarly exposed to dangerous situations and there is still a potential for the individual to be a victim or an offender in an ambiguous and threatening situation. Empirical research corroborates this theoretical assumption and the current findings, which note a disproportionate number of people with ID in the criminal justice system and suggest their particular susceptibility to sexual offending [2, 14, 33, 34]. In line with the Routine Activities Theory [17] the higher rates of offending may be related to the significant environmental and individual challenges faced by people with ID [2]. While offending may be more pronounced in people with ID, there is also a greater propensity for parental adversity, low socio economic status and mental illness. Future research should therefore seek to discern the relative importance of these variables to crime in ID populations.

### Comorbid mental illness

The presence of a co-occurring mental illness significantly increased the likelihood of people with ID having both victimisation and offending histories. Mental illness has been associated with victimisation and offending in other vulnerable populations [42–45] with one study suggesting that mental illness may be linked with criminality in people with intellectual disability [46]. The current findings suggest that mental illness complicates the association between ID and victimisation and offending considerably (almost doubling the rates of both).

The presence of a mental illness may further limit the functionality of the individual with ID and may intensify their exposure to dangerous situations and reactions to potential perpetrators. The association between ID with comorbid mental illness and increased rates of victimisation and offending indicates that treating mental illness alongside managing deficits associated with ID could have beneficial effects for crime prevention and victimisation. However, while mental illness appears to be pivotal in influencing susceptibility to crime, there may be other contributing factors such as substance abuse, outside of the scope of the present study, which further complicates the relationship.

The present study complements the existing literature base providing a robust contemporary prevalence estimate for victimisation and offending among people with ID. It adds further weight to prior research findings regarding criminality among those with ID and proposes that people with ID are at a significant disadvantage and are over represented in crime figures. By illustrating the magnitude of the problem, using a robust epidemiological design, it is hoped that there will be greater research into why this problem exists and how this effect can be minimised.

### Limitations

The findings in the current study may be limited by several factors that were inherent in the databases used. First, the Restrictive Interventions Database System (RIDS) may not be representative of all people with intellectual disabilities as individuals who are included on this reporting system have been subject to at least one restrictive intervention. This may have led to an over or underestimation of the true rates offending and victimisation reported here. While not available through the current dataset, taking into account a frequency criterion relating to the number of restrictive intervention episodes experienced by the person with ID as a potential confounding factor may help further develop our understanding of both risks and vulnerabilities to crime among people with ID who have more complex presentations or who present with more challenging behavioural management issues. Second, notwithstanding the challenges associated with diagnosing mental disorders among people with ID [47, 48], rates of mental illness were estimated from a public mental health database which, as noted in the methodology, under-reports some of the more high prevalence disorders. That being said, a strength with the current methodology was that case ascertainment of mental disorder and police contact for individuals in the community comparison sample was identical, thereby leading to direct comparability and greater confidence in the magnitude and direction of associations reported here. Thirdly, the findings may underrepresent the extent of crime involvement in ID as victimisation and offending data were based on contact with the police, where official reports were made and subsequently recorded. From the literature, we know that individuals with ID typically under report crimes and may find it difficult to recognise these crimes. It is likely that the current estimate of crime is more conservative compared to true prevalence figures [23]. Further, it was not possible to statistically control for the potentially confounding effect of age as the ID and community databases were independent of each other. While this is unlikely to affect the direction of the associations with violent and sexual offending, the younger age of the ID sample may explain the finding pertaining to their lower risk of being the victim of other types of non-sexual non-violent crimes found with this sample, although the evidence remains inconclusive. Lastly, the community comparison sample operationalized offending at the level of conviction, while the ID sample used the level of criminal charge; this may mean that the statistical differences presented may represent an upper confidence limit for estimates increased risks of both perpetration and victimisation histories.

### Directions for future research

Future research should seek to replicate and extend on current findings, which represent a preliminary yet robust insight into the vulnerabilities of those with ID. Of particular interest is future research is differences between specific age groups, mental illnesses and specific licit and illicit substances. Mental illness was a key factor in the association between intellectual disability and crime perpetration, the influence of specific mental illness on ID was not considered in the current study, as diagnoses are difficult to establish with comorbid ID. A future study with a greater focus on the robust assessment of mental illnesses and substance use could identify more specific disorders that are pertinent to both victimisation and offending; such information would be critical to informing both risk assessment and treatment planning.

## Conclusions

Results of the current study provide robust prevalence estimates indicating that, statistically speaking, people with intellectual disability are at greater risk of experiencing violent and sexual victimisation and more likely to violently and sexually offend than non-disabled people living in the community. Future research should seek to elucidate why these differences can and do exist and should account for other contributing factors that may influence this relationship.

## Abbreviations

ID: intellectual disability
LEAP: Law Enforcement Assistance Program
NHMRC: National Health & Medical Research Council
RIDS: restrictive interventions data system
VPCR: Victorian Psychiatric Case Register
